# Chemoprotective effects of plasma derived from mice of different ages and genders on ovarian failure after cyclophosphamide treatment

**DOI:** 10.1186/s13048-020-00735-3

**Published:** 2020-11-25

**Authors:** Soghra Bahmanpour, Eisa Moradiyan, Farzaneh Dehghani, Nehleh Zarei-fard

**Affiliations:** 1 Anatomy Department, School of Medicine, Shiraz University of Medical Sciences, Zand St., Shiraz, 7134845794 Iran; 2 Histomorphometry and Stereology Research Center, Shiraz University of Medical Sciences, Shiraz, Iran

**Keywords:** Ovarian failure, Plasma, Cyclophosphamide, Follicle, Stereology

## Abstract

**Background:**

Premature ovarian failure is one of the major side effects of chemotherapy drugs. Blood plasma contains several factors that might lead to the repair of different tissues.

**Objective:**

The chemoprotective effects of plasma derived from mice with different ages and genders were assessed on ovarian tissue in cyclophosphamide-treated mice.

**Methods:**

Forty-two adult female mice were divided into six groups as follows: (A) control; (B) 0.9% sodium chloride as vehicle; (C) cyclophosphamide; (D) cyclophosphamide + young male blood plasma; (E) cyclophosphamide + old male blood plasma; (F) cyclophosphamide + young female blood plasma. Ovarian failure was induced by injecting cyclophosphamide. On the 1st day, three groups received simultaneous injections of 150 μL intraperitoneal and 70 μL intravenous plasma derived from mice of different ages and genders. Each plasma type (150 μL) was then injected intraperitoneally every other 3 days for 19 days. On day 21, the dissected ovaries were stained for stereological analysis. Also, estrogen and progesterone levels were measured.

**Results:**

Cyclophosphamide had damaging effects on ovarian parameters and led to reduced hormone levels in comparison with the control group. However, treating with young female and, old male blood plasma, to a lesser degree, showed beneficial effects on the number of primordial follicles, pre-antral follicles, and granulosa cells. Also, these two treatments had protective effects on the volume of ovarian parameters as well as estrogen and progesterone levels in comparison with the cyclophosphamide group (*P* < 0.05).

**Conclusion:**

Plasma derived from mice of different ages and genders can ameliorate premature ovarian failure against the adverse effects of cyclophosphamide.

## Introduction

Premature ovarian failure (POF) is a complex heterogeneous disorder, characterized by the cessation of the menstrual cycle, reduced estrogen and progesterone levels as well as depletion of all types of ovarian follicles especially primordial follicles in women < 40 years. POF, occurring in about 1% of women 30–40 years old, leads to some complications including infertility and osteoporosis [1]. The underlying causes of POF may be genetic disorders such as Fragile X syndrome, Turner syndrome, autoimmune diseases, or exposure to toxins such as viruses, cigarette smoke, radiation therapy or chemotherapy [2–4]. Cyclophosphamide (CYC), as an alkylating agent, is one of the most common and effective chemotherapy drugs used to treat some diseases like Addison disease, systemic lupus erythematosus, systemic sclerosis, thyroiditis, vasculitis, and different types of cancer [5–7]. Although CYC is an effective treatment for such patients, it has various side effects on normal tissues due to its wide distribution in the tissues and permeability of the biological barriers. The reproductive toxicity in both genders is an example of the tissue-destructive effects of CYC. In several studies, it was shown that CYC led to ovarian toxicity by induction of different signaling pathways which are involved in the ovarian follicle apoptosis, resulting in an increase in the incidence of POF and infertility [8–10]. Ovarian protection from CYC-induced toxicities is one of the major concerns of researchers. Several studies have pointed to the role of antioxidants, mesenchymal stem cells, and platelet-rich plasma (PRP) in the reduction of CYC-induced ovotoxicity. Also, the protective roles of the co-administration of gonadotropin-releasing hormone (GnRH) agonists or other chemotherapy drugs such as imatinib with CYC on ovarian reserve have been reported previously [8, 10–14]. Although ovarian protection by GnRH agonist administration during chemotherapy treatment has been recommended by the American Society for Reproductive Medicine, its protective effects are controversial and not generalizable to all patients [14]. Further studies should, therefore, investigate other protective agents following chemotherapy.

Previous studies also showed that renal subcapsular grafting of aged ovarian tissues to young adult female mice could support oocyte production from quiescent germ cells in aged ovaries, which was due to young host blood contents [15]. Moreover, parabiotically sharing of the circulatory system of young female or aged male mice with normal young female mice increased primordial follicles [15, 16]. Young and old mice parabiosis or transfusion of young adult plasma to old mice also improved memory and learning in the old mice [17, 18]. Moreover, one of the routine therapeutic interventions in patients with malignancies, autoimmune diseases, or other disorders is the transfusion or exchange of plasma units alongside the other treatments [19, 20]. Plasma contains nutrients, transport proteins, hormones, and microparticles derived from various cells that make the plasma a rejuvenating factor [21, 22]. Nonetheless, there was no indication that plasma derived from different sources protected ovaries against chemotherapy-induced POF. Therefore, in the present study, we assessed the effects of young and old-derived plasma and also sex-mismatched plasma on CYC-induced ovarian failure in mice through a quantitative stereological analysis.

## Methods

### Animals

The experiments were designed on forty-two healthy BALB/c female mice (approximately, 28–30 g and 8 weeks old) purchased from the Center of Comparative and Experimental Medicine, Shiraz University of Medical Sciences, Iran. Animals were kept in normal conditions (temperature of 21 ± 2 °C and light/dark cycle of 12:12 h) with free access to food and water in standard cages. The mice were acclimatized for 2 weeks before the experiments were initiated. The study was approved by the local Ethics Committee of Shiraz University of Medical Sciences for animal experimental procedures.

### Collection of mice plasma from blood samples

To prepare the plasma, mice of different ages and genders were selected as follows: A, young males (2 months old); B, old males (14 months old); and C, young females (8 weeks old at the stage of late proestrus or early estrus cycle). To prevent stress, all the healthy animals were housed under standard conditions. For blood collection, the mice were anesthetized using chloroform and then immediately dissected. Approximately 2 mL of blood from each group was collected from the inferior vena cava and the heart; the blood was then immediately transferred into tubes containing ethylenediaminetetraacetic acid (1.5 mg/ml blood). Blood plasmas were prepared by centrifugation at 1200 rpm for 10 min using a refrigerated centrifuge; they were then stored at − 80 °C until injection [23].

### Experimental design

**Fig. 1:**
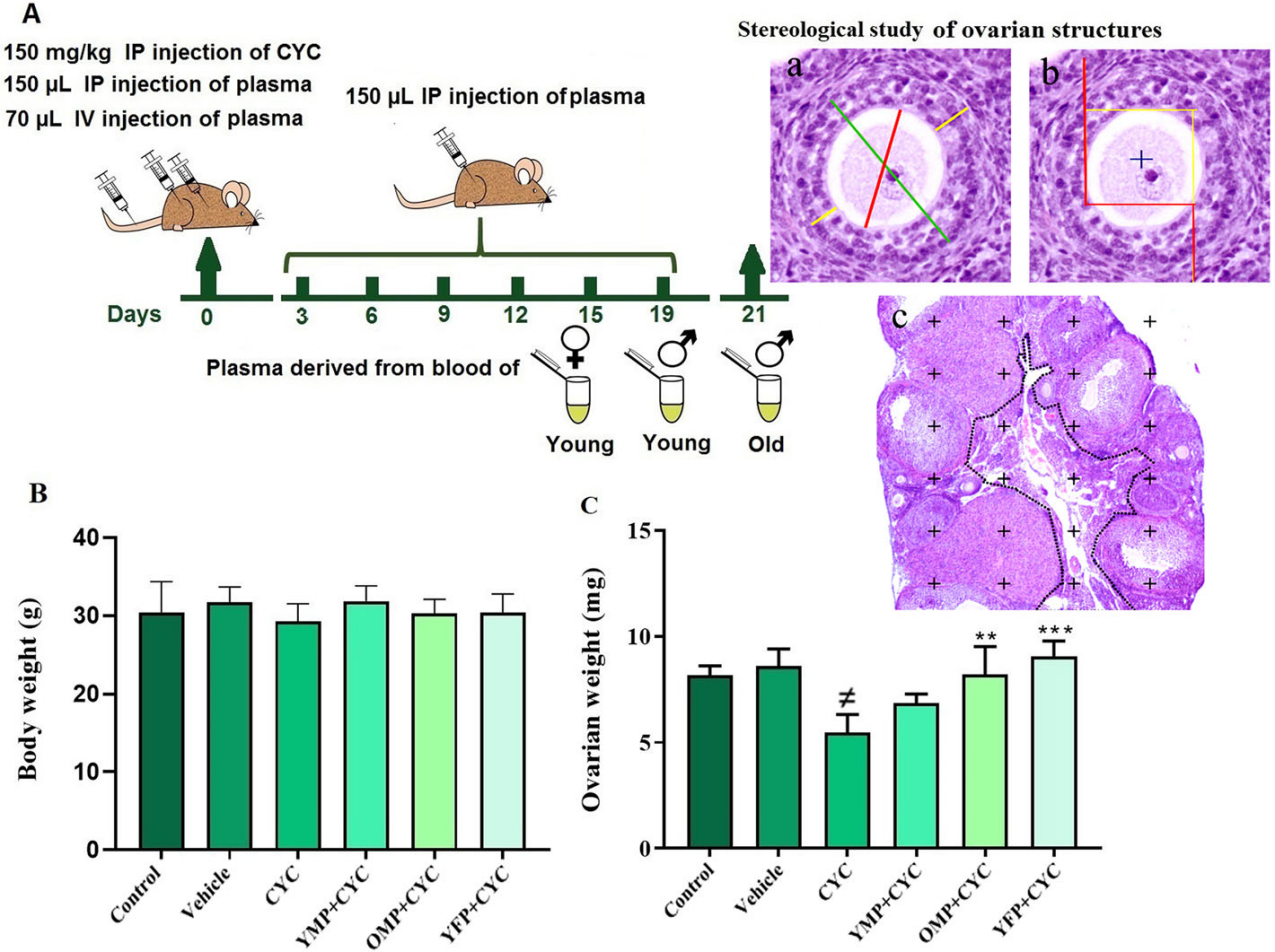
**A** Schematic representation of the experimental protocol used for the cyclophosphamide (CYC) -induced ovarian failure and treatment by plasma derived from old male plasma (OMP), young female plasma (YFP) and young male plasma (YMP) up to day 19. Histological sections show stereological methods. **a** Mean value of the 4 different diameters of oocyte, follicle and granulosa cells to estimate diameters, **b** Optical dissector method by unbiased counting frame on histological section to estimate number, **c** Cavalieri point*-*counting method to estimate the volume density of the ovarian structures with fraction of the ovary marked with points over histological section that also specifies the peripheral rim of cortex of the central medulla. **B** Analysis of animal weight and **C** ovarian weight in all groups. *and # indicate significant change from CYC and control respectively. Values are Mean ± SD; (* *P* < 0.05); (**P ≤ 0.001)

Mature healthy female mice were divided randomly into six groups of seven mice each (*n* = 7). The group distribution was as follows (Fig. 1A):

In the first day of experiment, group A animals received no treatment and served as the control. Group B animals received only 0.9% sodium chloride, 150 μL intraperitoneally (IP) as well as 70 μL intravenously (IV) in the tail and served as a vehicle control group. Group C was treated with a single dose of CYC 150 mg/kg, whereas groups D, E, and F received single dose of 150 mg/kg CYC (IP injection) along with 150 μL plasma (IP injection) and 70 μL plasma (IV tail injection) derived from blood of young male, old male, or young female mice, respectively. On the next days of the experiment, groups D, E, and F were treated with 150 μL of their respective blood plasma types (IP injection) every 3 days up to the 19th day. Group B, however, received only 0.9% sodium chloride every 3 days up to the 19th day.

On the 21st day of the experiment, mice were weighed and sacrificed by inhalation of ethyl ether. Immediately after dissection, the right ovaries were removed for fixation with neutral buffered formalin. After staining of the ovarian sections with hematoxylin and eosin (H&E), stereological assessments were performed. Also, for hormonal assay, serum samples were obtained by centrifugation of the collected blood clot samples at 1200 g for 10 min and kept at − 80 °C to prevent degradation.

An average of 10 histological slides per ovary was counted by serial sectioning (5 μm thin, 50 μm interval, and 25 μm thick sections) to estimate ovarian parameters using unbiased stereology. Slides with 5 μm or 25 μm thickness were used for volume and cell number estimation, respectively.

### Estimating the volume and diameter of ovarian structures by stereological study

This assessment was performed using the Cavalieri method. After staining with H & E, 10–12 sections were selected in a systematic random manner and examined using a video microscope at 2.4 magnification. The total ovarian volume and the ovarian structures were calculated by the point counting method using the following formula [24]:

$$ \mathrm{V}=\sum \mathrm{P}\times \left(\mathrm{a}/\mathrm{p}\right)\times \mathrm{t} $$

Where ‘∑P’ was the total number of points hitting the sections, ‘a/p’ was the area per point, and ‘t’ was the distance between the sampled sections. Additionally, ‘a/p’ was estimated by the following formula:

$$ \left(\mathrm{a}/\mathrm{p}\right)=\left(\varDelta \mathrm{x}\times \varDelta \mathrm{y}\right)/{m}^2 $$

Where ‘∆x’ and ‘∆y’ were the distances between the two adjacent points on the grid in the x-axis or the y-axis, respectively, ‘m’ was the final linear magnification of the microscopic images.

Diameters of follicles, oocytes, and granulosa cells were also calculated by measuring the mean value of 4 different diameters of each one by stereo lite software.

### Estimation of ovarian cell number by stereological study

Since various types of follicles were visible in each ovarian cycle, four types of ovarian follicles were considered during the stereological counting. Primordial follicles possessed an oocyte surrounded by a single layer of squamous granulosa cells. Pre-antral follicles contained prominent nucleus and were surrounded by one or more layers of granulosa cells without cavity or antrum between the granulosa cells. In the antral follicles, the oocytes were enveloped by granulosa cells with an antrum between the cells. The atretic follicles contain an irregular shape and pycnotic nucleus [25–27]. Different types of follicles were counted by optical dissecting method using 20 μm thick sections. In this method, the smallest and most visible parts of the cells were used to accurately count the follicles and granulosa cells. The total number of follicles were estimated by stereological software (Stereo. Lite, SUMS, Shiraz, Iran). The unbiased counting frame superimposed the images that were viewed on the monitor.

An average of 150–200 microscopic fields were selected in each ovary via a systematic random sample (SRS). The position of the first area was selected randomly outside the sections and the other areas were selected by moving the microscope stage in an equal interval along the x- and y-directions using a stage micrometer. For counting the structures, oil-immersion lens (60x) was used. The final magnification was 1540x using a microcator (Heidenhain MT-12, Traunreut, Germany) that measures the z-axis traveling. Any nucleolus in focus at the starting 5 μm plane was excluded. Any nucleolus that came into maximal focus within the next traveling 5 μm optical section (height or dissector) was selected if it laid in the counting frame or touched the inclusion border and did not touch the exclusion borders or the frame. The numerical density of the different follicles and granulosa cells were obtained using the following formula [24]:

$$ \mathrm{Nv}=\left[\sum \mathrm{Q}/\sum \mathrm{P}\times \left(\mathrm{a}/\mathrm{f}\right)\times \mathrm{h}\right]\times \left(\mathrm{t}/\mathrm{BA}\right) $$

Here, ‘∑Q’ was the total number of the counted cells; ‘∑p’ was the total number of the points superimposed on the selected fields; ‘a/f’ was the frame area in the true tissue scale; ‘h’ was the tissue thickness (20 μm) considered for counting; ‘t’ was section thickness; and BA was the microtome setting. The result of the equation was then multiplied by the total volume of the ovary to obtain the total number of the different follicles and granulosa cells. The total number of the ovarian follicles was calculated using the following formula [24]:

$$ \mathrm{N}=\mathrm{Nv}\times \mathrm{V} $$

Where ‘NV’ was the number density of ovarian structures, ‘V’ was the ovarian volume.

### Enzyme immunoassay for estrogen and progesterone

To measure serum estrogen and progesterone levels, we used enzyme immunoassay kit (Monobind Inc., Philadelphia) according to the manufacturer’s protocol. All the samples were analyzed in triplicate for statistical analysis.

### Statistical analysis

The statistical analysis and graph plotting were performed using GraphPad Prism *(*8.2.0*.,* GraphPad*.* Software*,* San Diego*,* CA*,* USA) by One-way ANOVA followed by Turkey’s post-hoc tests. Data are presented as mean ± standard deviation of mean (SD). *P* values < 0.05 were considered to be statistically significant.

## Results

### Body weight and ovarian weight

The mean body weight of the animals was analyzed at the end of the experiments. Although the mean body weight of the CYC-treated group had decreased compared to the other groups, no significant changes were detected among the groups (*P* ≥ 0.05, Fig. 1B).

A comparison of ovarian weight between groups showed that the ovarian weight of CYC- treated group was significantly reduced compared to the control (*P* = 0.011) and vehicle (*P* = 0.049) groups. Ovarian weight loss was to some extent protected in the young male blood plasma treated group compared to the CYC group, but this difference was not significant. Also, the ovarian weight of the groups treated with old male blood plasma (*P* = 0.032) and young female blood plasma (*P* = 0.023) had significantly increased compared to the CYC treated group (Fig. 1C).

### The volume of the ovary and different ovarian structures

**Fig. 2:**
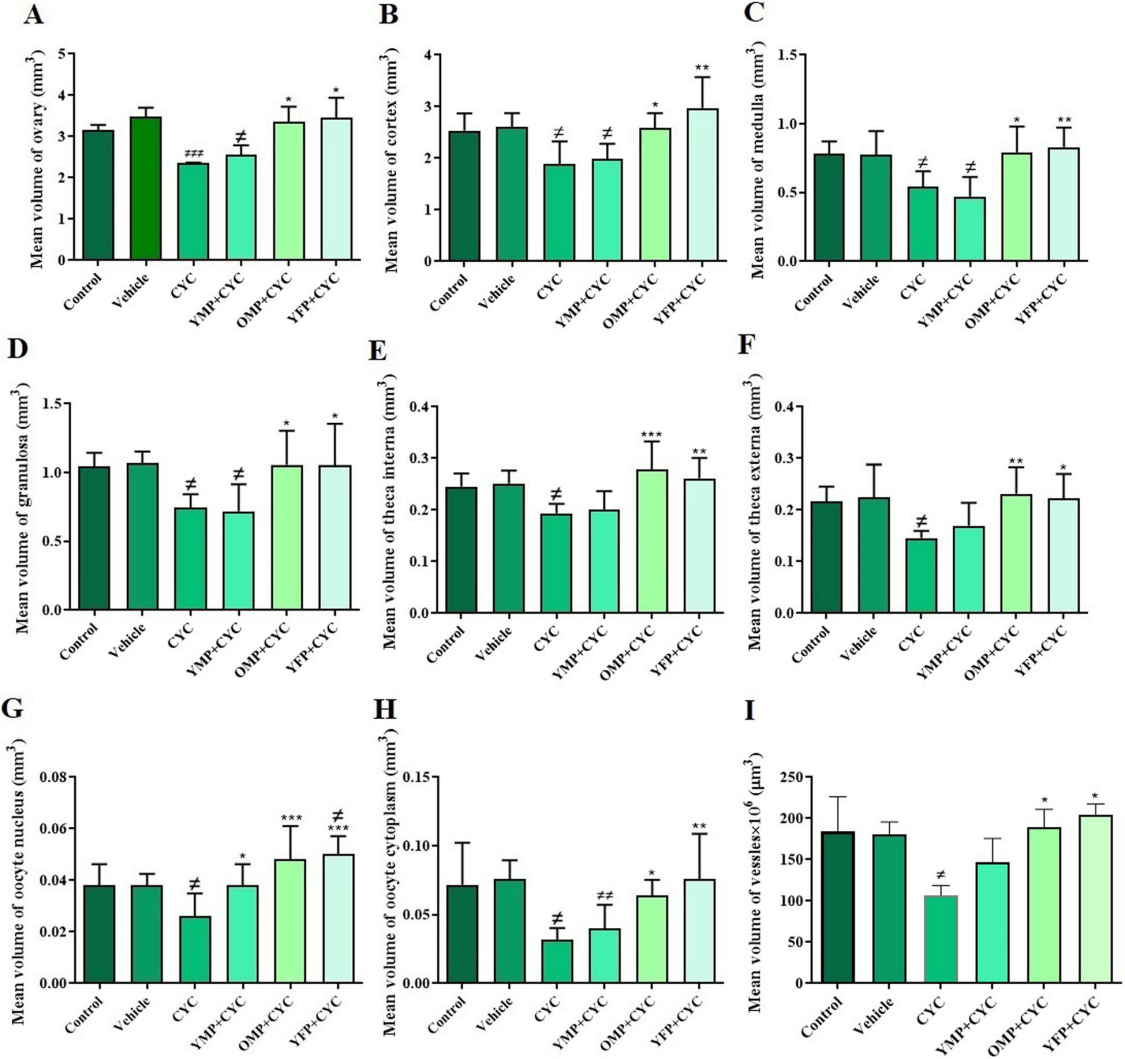
The effects of cyclophosphamide (CYC) and different origins of plasma on volume of different ovarian structures. Mean volume of ovary, cortex, medulla, blood vessels, nucleus and cytoplasm of oocytes, granulosa cells, theca interna and theca externa were determined by *stereological* analysis. The ovarian failure was induced by CYC injection and the mice were then treated with young male plasma (YMP), old male plasma (OMP), or young female plasma (YFP). *and # indicate significant change from CYC and control, respectively. Values are Mean ± SD; (*p < 0.05); (**P ≤ 0.01); (***P ≤ 0.001)

In this study, total ovarian volume, volume of ovarian cortex and medulla, nucleus and cytoplasm of oocytes, theca interna, theca externa cells as well as granulosa cells were calculated through stereological procedures (Fig. 2a-h). The results showed that the total ovarian volume in the CYC-treated group was significantly reduced compared to the control (*P* = 0.001) and vehicle (*P* = 0.0012) groups. Moreover, the ovarian volume was significantly increased in the young female (*p* = 0.033) and old male (*P* = 0.039) plasma-treated groups compared to the CYC-induced ovarian failure group (*P* = 0.011). On the other hand, in the group treated with young male plasma, total ovarian volume was higher than that in the group treated with CYC, but this difference was not significant (*P* = 0.17).

The mean volume of ovarian cortex (*P* = 0.014), medulla (*P* = 0.015), nucleus (*P* = 0.040) and cytoplasm of oocytes (*P* = 0.006), theca interna (*P* = 0.028) and theca externa (*P* = 0.020) layers, as well as granulosa cells (*P* = 0.021) in the CYC-induced ovarian failure was significantly reduced compared to the control group. However, the volume of those structures was significantly increased in the young female and old male plasma-treated groups compared to the CYC-induced ovarian failure (*P* < 0.05). Also, a significant increase in the volume of oocyte nucleus was observed in the young male (*P* = 0.041) plasma treated group while the other ovarian parameters had not changed compared to the group treated with CYC.

The total volume of blood vessels in the CYC-induced ovarian failure group was significantly declined in comparison with that of the control group (*P* = 0.041). On the other hand, the CYC-induced ovarian failure animals that were treated with young female and old male plasma did not experience a volume loss in blood vessels compared to the control and vehicle groups (*P* = 0.32 and *P* = 0.8, respectively) (Fig. 2i).

### Diameter of ovarian follicles, oocytes and granulosa cells

**Fig. 3:**
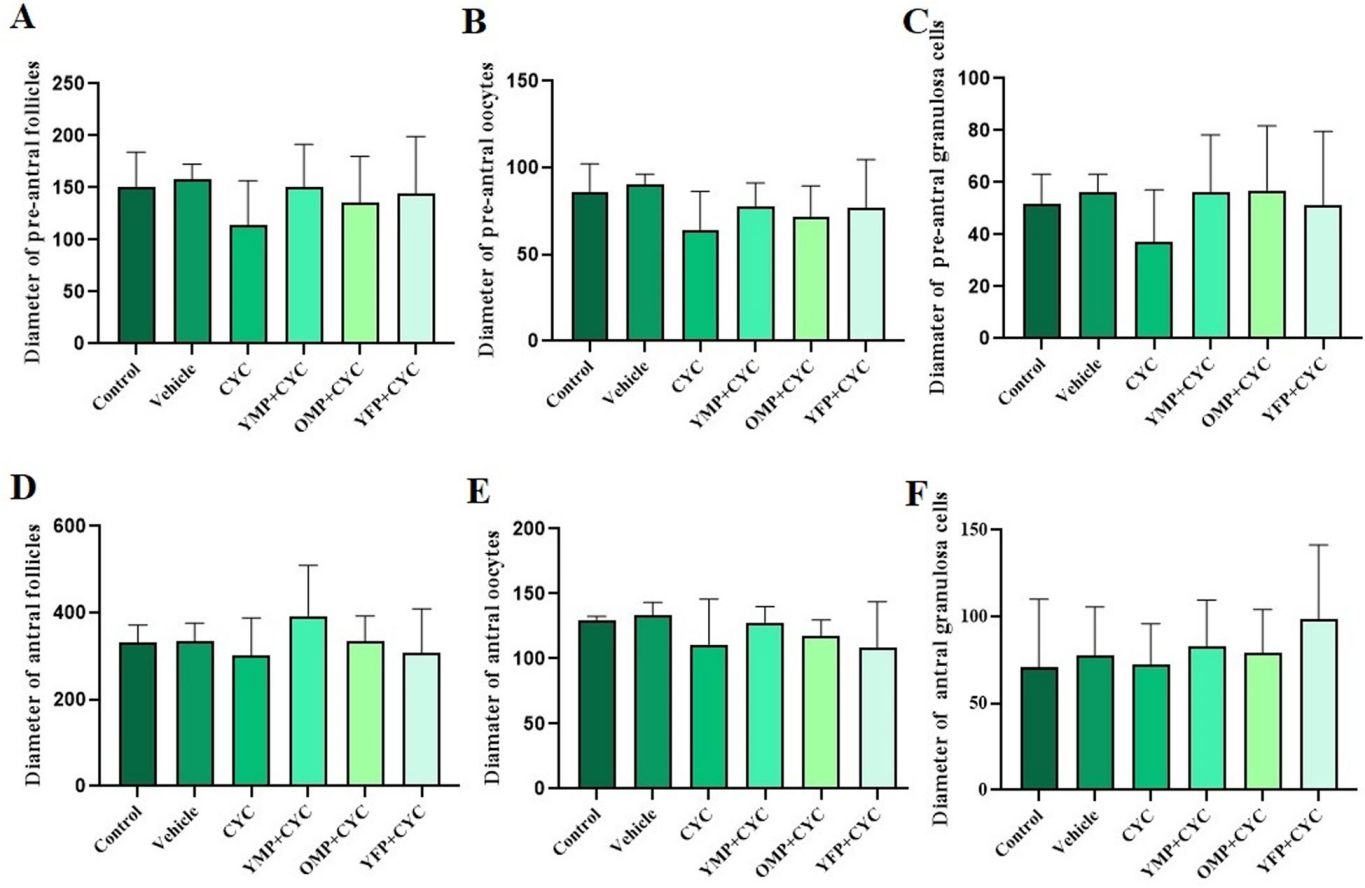
*Stereological* analysis of diameter of antral and pre-antral follicles as well as the diameter of oocytes and granulosa cells between the control, vehicle, cyclophosphamide (CYC)-, young male plasma (YMO)-, old male plasma (OMP)- and young female plasma (YFP) -treated groups. Values are Mean ± SD

A comparison of the diameter of the antral and pre-antral follicles as well as the diameter of the oocytes and granulosa cells in these follicles (Fig. 3. 1A-G) showed that the diameters in the CYC-induced ovarian failure were lower than those of the other groups, but these differences were not statistically significant.

### The number of ovarian follicles and granulosa cells

**Fig. 4:**
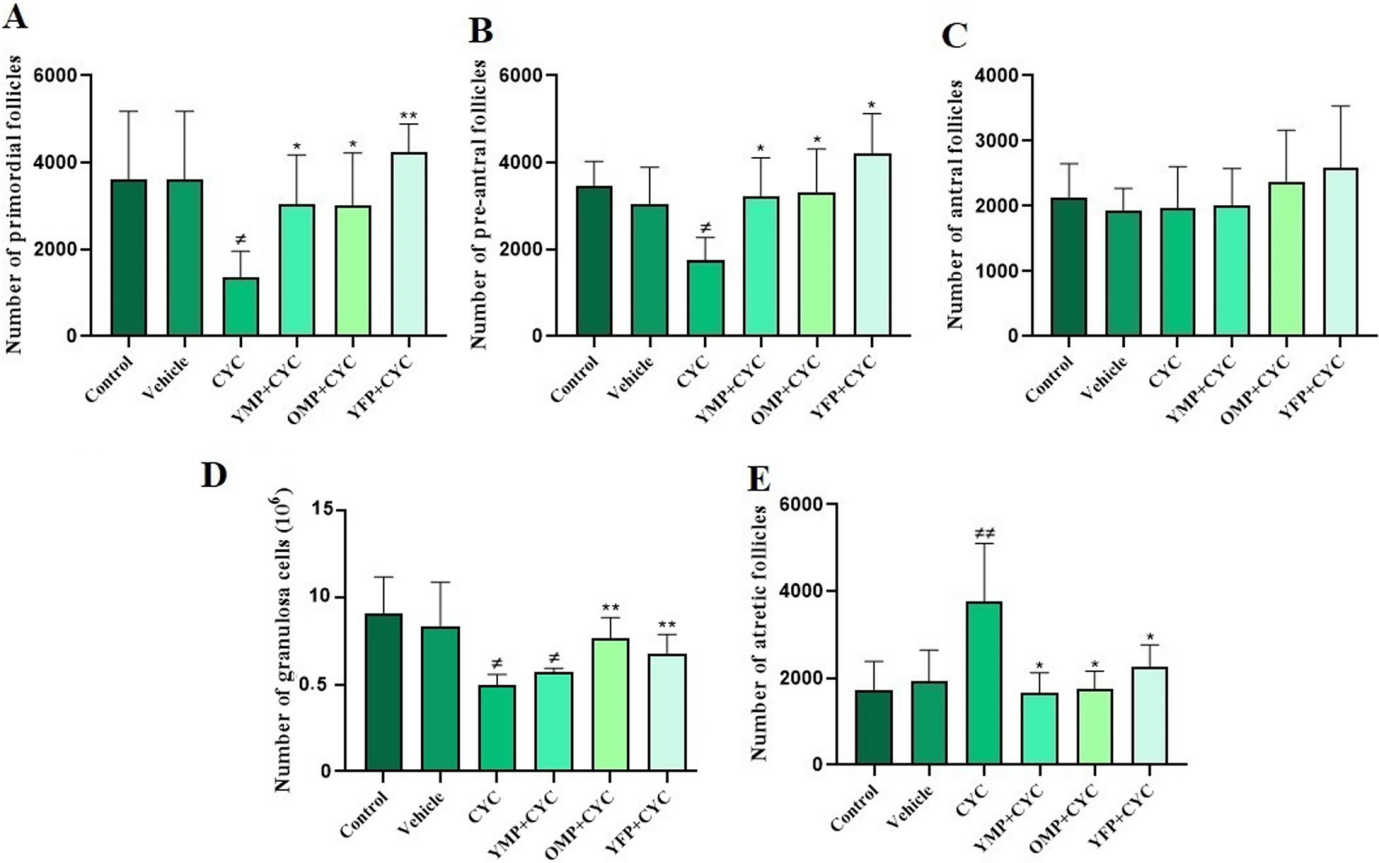
The mean number of morphologically normal cells in each ovary was determined by stereological analysis in six groups including control, vehicle, cyclophosphamide (CYC)- young male plasma (YMO), old male plasma (OMP) and young female plasma (YFP)- treated groups. *and # indicate significant change from CYC and control, respectively. Values are Mean ± SD; (*P < 0.05); (**P ≤ 0.001)

**Fig. 5:**
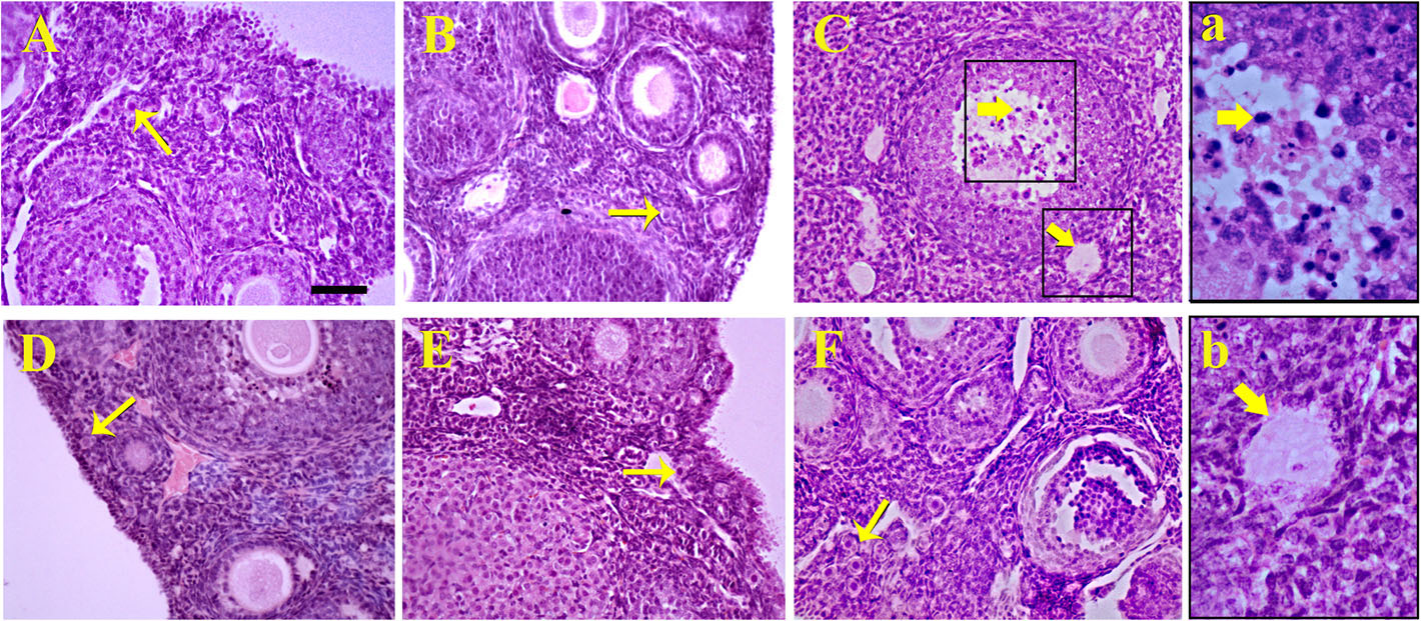
Hematoxylin and eosin staining of ovaries in **a** control, **b** vehicle, **c** cyclophosphamide (CYC)-induced ovarian failure and treatment by plasma derived from **d** young male plasma, **e** old male plasma, **f** young female plasma. Histology of ovaries showed (a and b) higher number of atretic follicles (thick arrows) in CYC group compared to the control, and also higher primordial follicle number (thin arrows) in the groups treated with old male and young female plasma compared to CYC-induced ovarian failure. Scale bar: 50 μm

The mean number of ovarian follicles in the experimental groups showed that the number of primordial follicles (*P* = 0.036) and pre-antral follicles (*P* = 0.011) in the CYC-treated group significantly reduced compared to that of the control group (Fig. 4). Figure 5 shows the histological section of ovarian follicles in all the designed groups. When the groups were compared to each other, there was a significant increase in the number of primordial and pre-antral follicles (Fig. 4a and b) after the treatment with the plasma derived from young males (*P* = 0.034 and *P* = 0.019), old males (*P* = 0.026 and *P* = 0.043), and young females (*P* = 0.004 and *P* = 0.014) in comparison to the group that had received CYC. However, no significant difference in the number of antral follicles (Fig. 4c) was found between different experimental groups. Also, the number of morphologically normal granulosa cells (Fig. 4d) in the CYC-induced ovarian failure group was significantly reduced compared to that of the control (*P* = 0.015) and vehicle-treated (*P* = 0.049) groups. The number of granulosa cells in the groups treated with old male (*P* = 0.0059) and young female plasma (*P* = 0.008) was significantly increased compared to the CYC-induced ovarian failure group. However, the number of granulosa cells in the group treated with young male plasma had slightly, but not significantly, increased. Moreover, the number of atretic follicles (Fig. 4e) in the group treated with CYC (*P* = 0.0046) had significantly increased compared to that of the other experimental groups although there was no significant difference in the number of atretic follicles between the plasma-treated groups and the control and vehicle groups.

### Measurement of estradiol and progesterone

**Fig. 6:**
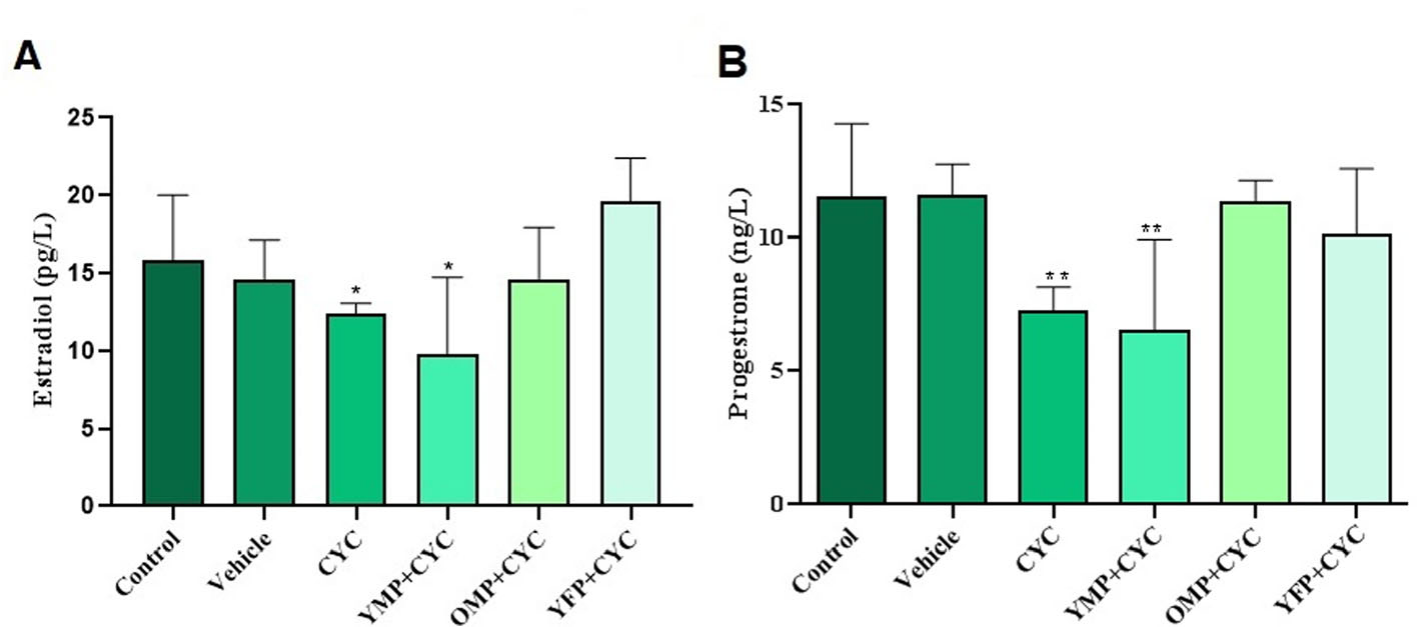
Estradiol and progesterone concentrations in blood serum in different experimental groups after cyclophosphamide (CYC)-induced ovarian failure and treatment with plasma derived from old male plasma (OMP), young female plasma (YFP) and young male plasma (YMP). * indicate significant change from control. Values are Mean ± SD; (* *P* < 0.05)

Analysis of the CYC-induced ovarian failure showed a significant decrease in the estradiol and progesterone concentrations compared to the concentration of the control (*P* < 0.05, Fig. 6). Serum progesterone concentrations were significantly elevated in the young female and old male plasma - treated groups compared to CYC-induced ovarian failure group (*P* = 0.01 and *P* = 001, respectively). When estradiol concentrations were analyzed, the highest levels of this hormone were found in the mice treated with young female and old male plasma, respectively. In contrast, young male plasma-treated group showed lower levels of estradiol than the other groups.

## Discussion

Cyclophosphamide, as a commonly used chemotherapy drug, is effective in treating many patients suffering from a wide range of diseases including cancers and autoimmune complications. However, it has deleterious side effects on some organs, most notably the reproductive system [5–7]. Several studies showed that many patients who were exposed to CYC had experienced POF [28–30]. It is recognized that morphological changes and toxic effects of CYC on ovary occur through both death receptor and mitochondrial apoptotic pathways. Researchers have shown that CYC leads to DNA double strand breaks in oocytes and then cell demise occurs through activation of signaling axes including DNA-dependent protein kinase and ataxia telangiectasia mutated / checkpoint kinase 2 / p53 and alpha TAp63 isoform (TAp63α) and protein kinase B (AKT)/forkhead box O3 (FOXO3a) [10]. Besides, CYC induces apoptotic pathways by generation of oxidative stress and, as a result, release of cytochrome c from mitochondria into the cytosol and binds to apoptotic protease activating factor-1 (APAF-1) and also cleaved caspase-3 formation [31, 32]. Undoubtedly, inhibition of CYC-induced DNA damage can protect ovaries from apoptosis. Previous studies demonstrated that endogenous and exogenous contents of plasma could protect some tissues after experimental injuries, by activation of phosphatidylinositol 3-kinase (PI3K) /AKT/ mammalian target of rapamycin and mitogen-activated protein kinase signaling pathways involving in the transmission of anti-apoptotic signals in cells [33–35].

In this study, the effects of plasma derived from mice of different ages and genders against the toxic and destructive effects of CYC on the ovaries of adult mice were investigated. Our stereological studies showed that even though CYC administration did not have a significant effect on the animals’ weight, it could reduce various parameters such as ovarian weight, mean volume of entire and various parts of the ovaries, number of normal granulosa cells and different follicles (primordial, pre-antral) compared to the same parameters in the control group. Also, CYC increased the number of atretic follicles compared to the that of the control group. There are several reports on the depletion of primordial and pre-antral/antral follicles which is due to the direct and indirect effects of CYC on the induction of cell death in oocytes and granulosa cells, respectively [3, 31]. The primordial and pre-antral follicles are more sensitive and prone to atresia than the antral and graffian follicles [27, 36]. Moreover, CYC can induce growth arrest in granulosa cells, which plays a significant role in the nutrition and protection of oocytes and ultimately the maturation of oocytes. It was stated that these cells, especially mitotic ones, are the main target of chemotherapy drugs [27, 36, 37]. Chemotherapy can reduce ovarian follicle pool through vascular damage [3, 38]. It seems that the blood vessel damage and ultimately ischemia in the ovaries after CYC exposure, which we also found in our study, lead to the atresia of the follicles. It has been proposed that ovarian protective drugs, before and during chemotherapy, are able to preserve the fertility in young adult female cancer survivors. One fertility preservation technique is the administration of GnRH agonists which has been used to reduce ovarian toxicity during chemotherapy [39, 40]. However, there are some controversies over the protective effects of GnRH administration on CYC-induced POF [41]. Plasma transfusion is recommended for patients with cancer especially those who undergo surgical procedures [19, 20]. To determine whether plasma treatment could protect ovaries from damages, we injected young and old mice-derived plasma which were also sex-mismatched in mice with ovarian failure induced by CYC for 19 days. As another important finding, we determined that plasma treatment, especially young female and old male plasma, prevented primordial follicle, pre-antral follicle, and granulosa cell depletion. A previous study has shown that exposure to aged male mice blood after parabiotic joining can increase the number of primordial follicles in old females and, as a result, has rejuvenating effects. In contrast, young female or male mice blood was reported to have no beneficial effect on increasing primordial follicle pool [15, 16]. Effectiveness of young plasma transfusion or parabiotic joining in rejuvenation of old organ function is proven by several studies [17, 18, 42]. In fact, the type and level of circulating factors such as growth factors, hormones, metabolites, and cytokines in plasma, as non-cellular components of blood, are associated with age- and gender-related differences [43–45]. To exemplify, both follicle*-*stimulating hormone (FSH) and luteinizing hormone (LH) levels increase in aged males [46]. The higher levels of FSH promoted oocyte growth and ovarian follicular development and caused the secretion of estradiol in the developing follicles [47]. Moreover, LH and FSH have anti-apoptotic effects on oocytes and granulosa cells [48]. LH could protect ovaries from cisplatin-induced toxicities by activation of cyclic AMP/protein kinase A and PI3K/AKT pathways. Activation of these pathway prevent apoptosis by reduction of TAp63 levels and initiate DNA repair mechanisms in the oocytes [35]. Besides, FSH treatment inhibit granulosa cell apoptosis by suppression of reactive oxygen species production, cytochrome c/APAF-1/caspase-9, and AKT/FOXO3a signaling pathways [49, 50]. Besides, the levels of testosterone and dihydrotestosterone in male mice plasma are negatively correlated with age. However, the decrease of serum androgen levels in old male is still higher compared to young female mice [45]. In rodents, in-vivo administration of androgens can induce signaling pathways and FSH receptor expression in the ovary and, as a result, prevent apoptosis and promote follicle development [49, 51, 52]. Nevertheless, high concentrations of testosterone can disturb late stages of follicle development and stimulate granulosa cell apoptosis [53]. Therefore, the supportive effects of old male plasma on CYC treatment may be attributed to the influence of the hormonal contents that play an essential role in regulation of follicular survival and development.

The present experiment also showed that there was a reduction in estradiol and progesterone levels after CYC administration. In contrast, the plasma therapy significantly increased the levels of estradiol and progesterone in young female and old male plasma-treated groups compared to only CYC- treated group. Estradiol and progesterone hormones are produced in the granulosa cells of the growing follicles [27]. Therefore, reduction of granulosa cells in the growing follicles is the main reason for the drop in the levels of estrogen and progesterone in the cyclophosphamide-treated group compared to the control group. After plasma treatment, hormone levels were improved, which could be due to the effective role of plasma injection in protecting granulosa cells against the damaging effects of CYC chemotherapeutic agent. Moreover, our results revealed an increase in the number of and the volume of granulosa cells in young female plasma-treated group compared to that of the CYC-treated group. However, increase in the volume of granulosa cells was higher than increase in their number. Also, no statistically significant elevated estradiol and progesterone levels was found in young female plasma- treated groups compared to the respective control group. This might be due to the differentiation of small non-steroidogenic granulosa cells into large cells with the ability to secrete both estradiol and progesterone [54].

## Conclusion

Our preclinical findings suggest that plasma injection has protective effects on CYC-induced ovarian failure, age and gender dependently. Therefore, in treatment with plasma transfusion during CYC therapy, the age and gender of the plasma donor is recommended to be considered. However, clinical data should further confirm the efficacy of plasma administration in the reduction of ovarian damages caused by chemotherapy. In this study, the most effective choices were the transfusion of young female plasma and old male plasma, respectively. Further research is yet warranted to better understand this mechanism of action.

## Data Availability

All data generated or analyzed during this study are included in this published article.

## Abbreviations

POF: Premature ovarian failure
CYC: Cyclophosphamide
IP: Intraperitoneally
IV: Intravenous
H&E: Hematoxylin and Eosin
LH: Luteinizing hormone
FSH: Follicle-stimulating hormone
OMP: Old male plasma
YFP: Young female plasma
YMP: Young male plasma
